# Time and time again: unisexual salamanders (genus *Ambystoma*) are the oldest unisexual vertebrates

**DOI:** 10.1186/1471-2148-10-238

**Published:** 2010-08-03

**Authors:** Ke Bi, James P Bogart

**Affiliations:** 1 Department of Integrative Biology, University of Guelph, Guelph, Ontario, N1G 2W1 Canada; 2 Museum of Vertebrate Zoology, University of California, Berkeley, California 94720 USA

## Abstract

**Background:**

The age of unisexual salamanders of the genus *Ambystoma *is contentious. Recent and ancient evolutionary histories of unisexual *Ambystoma *were proposed by a few separate studies that constructed phylogenies using mitochondrial DNA markers (cytochrome b gene vs. non-coding region). In contrast to other studies showing that unisexual *Ambystoma *represent the most ancient unisexual vertebrates, a recent study by Robertson et al. suggests that this lineage has a very recent origin of less than 25,000 years ago.

**Results:**

We re-examined the phylogenetic relationship of the unisexuals to *A. barbouri *from various populations using both mitochondrial markers as well as the complete mitochondrial genomes of *A. barbouri *and a unisexual individual from Kentucky. Lineage dating was conducted using BEAST and MultiDivTime on a complete mitochondrial genome phylogeny. Our results support a monophyletic lineage for unisexual *Ambystoma *that shares its most recent common ancestor with an *A. barbouri *lineage from western Kentucky. In contrast to the Robertson et al.'s study, no *A. barbouri *individual shared an identical or almost identical cytochrome b haplotype with any unisexual. Molecular dating supports an early Pliocene origin for the unisexual linage (~5 million years ago). We propose that a unisexual-like cytochrome b *numt *(or pseudogene) exists in the controversial *A. barbouri *individuals from Kentucky, which was likely the cause of an erroneous phylogeny and time estimate in Robertson et al.'s study.

**Conclusion:**

We reject a recent origin of unisexual *Ambystoma *and provide strong evidence that unisexual *Ambystoma *are the most ancient unisexual vertebrates known to exist. The likely presence of an ancient cytochrome b *numt *in some Kentucky *A. barbouri *represents a molecular "fossil" reinforcing the hypothesis that these individuals are some of the closest extant relatives to unisexual *Ambystoma*.

## Background

Because of the absence of sex, a brief evolutionary lifespan is generally expected for unisexual and asexual organisms [1]. Nevertheless, ancient unisexuals and asexuals that persist millions of years have been discovered in various taxa among plants, fungi and animals [2]. With recent advances in molecular genetics and phylogenetics, our knowledge of reproductive systems and evolutionary histories of many unisexual and asexual lineages has been quickly improved. Recent evidence reveals that many unisexuals are capable of utilizing modified reproductive modes and/or mitotic or meiotic mechanisms to incorporate "a bit of sex" [3-7], or they can incorporate additional genetic material to compensate for the suspected lethal effects caused by the accumulation of deleterious mutations [7-10]. Thus, although populations may be all female, unisexual are not necessarily equivalent to asexual populations [5]. The discovery of ancient unisexual and asexual lineages not only poses a dilemma for evolutionary theoreticians but also provides an opportunity to address questions that relate to the prevalence and maintenance of sexual reproduction.

Unisexuality in vertebrates has been discovered in about 90 lineages of fresh water fish, amphibians and reptiles [11], most of which are recently spun off from sexual relatives via interspecific hybridization [7,12]. North American unisexual mole salamanders of the genus *Ambystoma *co-evolve with five distinct sexual ambystomatids (*A. laterale, A. jeffersonianum, A. tigrinum, A. texanum*, and *A. barbouri*) across the entire unisexual distribution [9,13-15]. Unisexual *Ambystoma *persist as a "parasitic entity" by stealing and incorporating sperm from sympatric sexual species via a complex reproductive mode, kleptogenesis, to generate nearly 30 genomic combinations or biotypes, with ploidy levels ranging from diploid to pentaploid [5,15]. Genomes in unisexuals may not be transmitted unaltered. Recent studies using genomic *in situ *hybridization (GISH) demonstrate complex intergenomic exchanges in unisexual populations [4,16-18].

Despite the complexity of their nuclear genomes, all unisexuals contain a highly conserved mitochondrial genome which is derived from *A. barbouri*, suggesting that *A. barbouri *may be the maternal ancestor of the unisexual lineage [5,9,19]. Although the ancestry of unisexual *Ambystoma *is not contested by these studies, the age of the unisexual lineage is controversial. Unisexual *Ambystoma *have been proposed as the most ancient unisexual vertebrates known to exist [5,20,21]. Based on a phylogeny constructed by the mitochondrial intergenic spacer and control region (mitochondrial non-coding region or NCR), Bogart et al. [5] estimated that the unisexual lineage and the closest relatives, an *A. barbouri *lineage from Kentucky, are descended from the most recent common ancestor 2.4-3.9 million years ago (Ma). Their results are based on the observation of a 3.91% pairwise difference in the control region between unisexuals and a few Kentucky *A. barbouri *individuals. On the contrary, Robertson et al. [19] constructed a phylogeny using a different mitochondrial marker cytochrome b (cyt-b) gene and suggested that unisexual *Ambystoma *have a very recent origin which could be less than 25,000 years ago. Strikingly, they found a few *A. barbouri *specimens that were also used by Bogart et al. [5] to have an identical or almost identical cyt-b haplotype to unisexual individuals. Such a large discrepancy is difficult to understand because the two different mitochondrial DNA markers (NCR vs. cyt-b) that came from the same *A. barbouri *individuals demonstrated distinctly different evolutionary relationships to the unisexual lineage (~3.91% vs. ~0%). Therefore, the question: "are unisexual *Ambystoma *ancient or recent?" is unresolved.

To provide a clearer answer to this question, we re-examined the phylogenetic relationship of unisexuals and *A. barbouri *from various populations using both mitochondrial cyt-b gene and NCR as markers. We especially focused on *A. barbouri *samples (JPB34337, JPB34342, JPB34343, JPB34356) that demonstrated distinctly different sequence divergences to the unisexuals in the two studies [5,19]. Given that the rate of substitution likely varies for different genes/regions in the mitochondrial genome, we compared the substitution rates of complete mitochondrial genomes (mtgenomes) of unisexual and *A. barbouri. *We sequenced the mtgenome of a Kentucky *A. barbouri *specimen (JPB34342) that was used in both studies and provided conflicting results. We chose to sequence the mtgenome of one tetraploid unisexual *A. laterale *- 3 *jeffersonianum *(or LJJJ) from a northern Kentucky population as a representative unisexual. This individual was chosen because it was found to have the most common mitochondrial NCR haplotype among unisexuals (haplotype B in [5]), it was collected in a state (Kentucky) where unisexuals were previously unknown, and it was geographically close to the *A. barbouri *individuals that were deemed to be the closest relatives to unisexual *Ambystoma*. For comparison, we also sequenced mtgenomes of one *A. barbouri *and one *A. texanum *from Ohio to examine their phylogenetic relationships to the Kentucky unisexual and *A. barbouri *individuals. The time to the most recent common ancestor (TMRCA) for unisexual lineage and its closest relative was re-calculated based on the mtgenome phylogeny.

## Results

### Mitochondrial cyt-b and NCR trees

**Figure 1:**
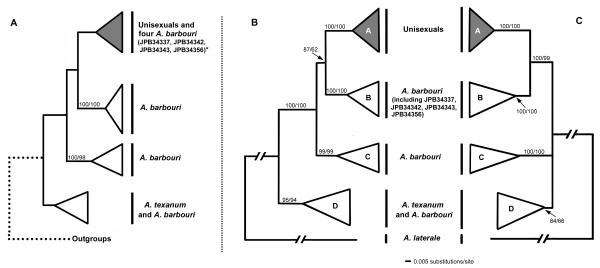
**Bayesian gene trees of mitochondrial cytochrome b (cyt-b) gene from Robertson et al. **[19]**[A] and the present study [B], and Bayesian gene tree of non-coding region (NCR) by the present study [C]**. The tree arrangement from Robertson et al. [19] has been slightly modified. Left numbers along branches represent the Bayesian posterior probabilities (BPP) and right numbers represent bootstrap proportions (BSP) derived from the parsimony analysis. The detailed information of specimens that were sequenced is listed in Table 1. *A unisexual-like cyt-b *numt *likely exists in *A. barbouri *specimens JPB34337, JPB34342, JPB34343 and JPB34356 [A] so they were grouped within the unisexual clade.

Primers Glu14100L, MLM651 and M2R (abbreviated as GMM) were used to amplify and sequence a ~ 2200 base pairs (bp) mitochondrial fragment that included cyt-b and NCR. A total of 46 cyt-b sequences, including one from an outgroup species (*Ambystoma laterale*) downloaded from GenBank, were compared (Table 1). Of the 1141 bp resolved, 228 sites were variable and 143 sites were phylogenetically informative. A total of 42053 most parsimonious trees resulted from the parsimony analysis with 335 steps, a consistency index (CI) of 0.758 and a retention index (RI) of 0.939. For NCR, 46 sequences including one *A*. *laterale *from GenBank were recovered (Table 1). Of the 1065 bp resolved, 215 sites were variable and 111 sites were phylogenetically informative. A total of 90 most parsimonious trees were obtained from the parsimony analysis with 310 steps, a CI of 0.768 and a RI of 0.944. For both markers, a GTR+I+G model was selected as the best-fit model. The cyt-b strict consensus tree (not shown) was nearly identical to the Bayesian tree, but NCR strict consensus (not shown) and Bayesian trees yielded slightly different topologies. The Bayesian trees with the BPP and BSP from the parsimony analyses are presented in Figure 1.

**Table 1:** Specimens used for phylogenetic construction by mitochondrial cyt-b and NCR in this study with GenBank accession numbers.

Clade	Vouchers (JPB)	Species or biotype*	Locality	GenBank No.
				
				cyt-b, NCR
A	11312	LTT	Pelee, ONT Essex Co.	GU078473, GU078514
	30066	LLJ	NJ Sussex Co.	GU078475, GU078516
	31283	LLJ	PA McKean Co.	GU078476, GU078517
	32232	LLJ	NY Niagara Co.	GU078477, GU078518
	37103	LLJ	MI Cass Co.	GU078507, GU078548
	37107	LLLJ	MI Cass Co.	GU078508, GU078549
	37128	LLJ	MI Cass Co.	GU078509, GU078550
	37816	LLJ	QUE Mirabel Co.	GU078510, GU078551
	**39932**	LJJJ	KY Kenton Co.	GU078472
B	32518	*A. barbouri*	KY Anderson Co.	GU078478, GU078519
	32519	*A. barbouri*	KY Anderson Co.	GU078479, GU078520
	32521	*A. barbouri*	KY Anderson Co.	GU078480, GU078521
	34326	*A. barbouri*	KY Oldham Co.	GU078481, GU078522
	34337	*A. barbouri*	KY Jessamine Co.	GU078488, GU078529
	34339	*A. barbouri*	KY Jessamine Co.	GU078489, GU078530
	34341	*A. barbouri*	KY Oldham Co.	GU078490, GU078531
	**34342**	*A. barbouri*	KY Oldham Co.	GU078469
	34343	*A. barbouri*	KY Oldham Co.	GU078491, GU078532
	34344	*A. barbouri*	KY Oldham Co.	GU078492, GU078533
	34355	*A. barbouri*	KY Mercer Co.	GU078496, GU078537
	34356	*A. barbouri*	KY Mercer Co.	GU078497, GU078538
	34357	*A. barbouri*	KY Mercer Co.	GU078498, GU078539
	34365	*A. barbouri*	KY Jessamine Co.	GU078502, GU078543
C	22765	*A. barbouri*	OH Montgomery Co.	GU078474, GU078515
	34327	*A. barbouri*	KY Franklin Co.	GU078482, GU078523
	34328	*A. barbouri*	KY Franklin Co.	GU078483, GU078524
	34331	*A. barbouri*	KY Fayette Co.	GU078484, GU078525
	34332	*A. barbouri*	KY Fayette Co.	GU078485, GU078526
	34333	*A. barbouri*	KY Fayette Co.	GU078486, GU078527
	34334	*A. barbouri*	KY Jessamine Co.	GU078487, GU078528
	39346	*A. barbouri*	OH Warren Co.	GU078512, GU078553
	34348	*A. barbouri*	OH Warren Co.	GU078493, GU078534
	39349	*A. barbouri*	OH Warren Co.	GU078513, GU078554
	34350	*A. barbouri*	OH Warren Co.	GU078494, GU078535
	34359	*A. barbouri*	KY Franklin Co.	GU078499, GU078540
	34360	*A. barbouri*	KY Franklin Co.	GU078500, GU078541
	34364	*A. barbouri*	KY Jessamine Co.	GU078501, GU078542
	34366	*A. barbouri*	KY Jessamine Co.	GU078503, GU078544
	34368	*A. barbouri*	KY Livingstone Co.	GU078504, GU078545
	34369	*A. barbouri*	KY Livingstone Co.	GU078505, GU078546
	**37710**	*A. barbouri*	OH Hamilton Co.	GU078470
	38876	*A. barbouri*	OH Butler Co.	GU078511, GU078552
D	34353	*A. barbouri*	TN Rutherford Co.	GU078495, GU078536
	34553	*A. texanum*	OH Clarke Co.	GU078506, GU078547
	**37892**	*A. texanum*	OH Montgomery Co.	GU078471
Out-group	-	*A. laterale*	-	NC_006330

Voucher numbers in bold represent specimens whose mtgenomes were sequenced.

* All unisexuals have at least one *A. laterale *[L] nuclear genome. The unisexuals used in the present study also have *A. jeffersonianum *[J] or *A. texanum *[T] nuclear genomes.

Both cyt-b and NCR trees clustered all specimens into four clades (Figure 1). All the unisexuals, irrespective of their biotypes and ploidy levels, formed a monophyletic clade A. Clade A was sister to clade B that contained *A. barbouri *individuals from southern Ohio and west of the Kentucky Rivers in central Kentucky. This clade corresponded to the *A. barbouri *"western clade" in Niedzwiecki's study [22]. Clade C contained *A. barbouri *from north or east of the Kentucky River and north of the Ohio River, as well as the disjunct populations in western Kentucky. This clade corresponded to the "northern clade" that was identified by Niedzwiecki [22]. Clade D included *A. texanum *and *A. barbouri *from Tennessee. In the NCR tree, the relationship of clade D in respect to three other clades was, however, not well resolved but the monophyly of clade A+ clade B was strongly supported (BSP = 99; BPP = 100).

In Robertson et al.'s study [19], four specimens (JPB34337, JPB34342, JPB34343, JPB34356) of *A. barbouri *were found to be clustered in the unisexual clade and three of them (JPB34337, JPB34342, JPB34343) shared the same cyt-b haplotype with some of the unisexuals (Figure 1A). We sequenced the cyt-b gene from the very same individuals but we found that none of them shared an identical or almost identical haplotype to any unisexual. We examined the sequence chromatogram of cyt-b in these individuals and only single signal peaks were observed at every site. All four samples were grouped in clade B with other *A. barbouri *where the average cyt-b sequence divergence between *A. barbouri *and unisexuals was 5.16% (Figure 1B). Likewise, no *A. barbouri *shared the same or even a very similar NCR sequence with unisexuals (Figure 1C), and the ingroup relationship in the NCR tree was concordant with that obtained by Bogart et al. [5].

### Amplification of cyt-b gene by MVZ15 and MVZ16

**Figure 2:**
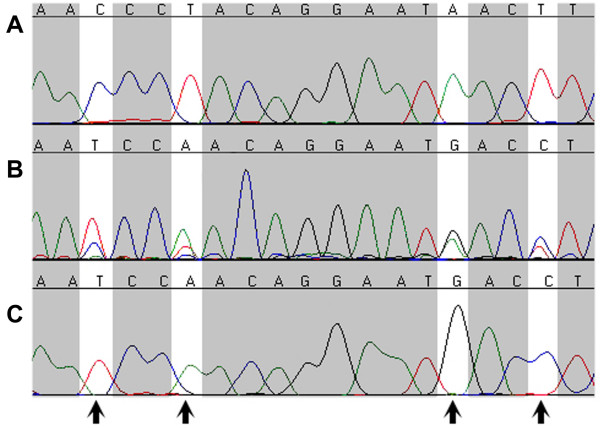
**Cyt-b fragment chromatograms showing the presence of unisexual-like cyt-b *numt *in *Ambystoma barbouri *individual JPB34356**. [A] Amplification and sequencing of cyt-b from JPB34356 using primers GMM, or using MVZ15/16 with mitochondrial DNA as template. [B] Amplification and sequencing of cyt-b from JPB34356 using primers MVZ15/16 with whole genomic DNA as template. [C] Amplification and sequencing of cyt-b in unisexual individual JPB30066, either using primers GMM or MVZ15/16 with whole genomic DNA as template. Arrows point to four sites that differ between *A. barbouri *and unisexual *Ambystoma*.

Bogart et al. [5] used primers MLMTHR and MLM651 to obtain a ~1100 bp intergenic spacer and control region from unisexual and *A. barbouri *specimens. We compared our sequencing results using the same primers and confirmed that MLMTHR and MLM651 produced the identical sequences to those by using primers GMM. Primers MVZ15 and MVZ16 were used by Robertson et al. [19] to amplify an ~800 bp cyt-b fragment from ambystomatids. In their study, *A. barbouri *individuals JPB34337, JPB34342, JPB34343 and JPB34356 were found to contain an identical or almost identical haplotype to unisexuals which placed them in the unisexual clade. Using the same primers, we failed to duplicate the same sequencing results in JPB34342 and JPB34343 as discovered by Robertson et al. [19]. Cyt-b failed to amplify in JPB34337 when using MVZ15 and MVZ16 but primers GMM confirmed that JPB34337 did not contain a unisexual haplotype. The results showed that these three individuals shared *A. barbouri *cyt-b haplotypes that were grouped with other *A. barbouri *in clade B (Figure 1B). We found, however, that JPB34356 did have a unisexual-like cyt-b haplotype as was found by Robertson et al. [19] using MVZ15 and MVZ16 as PCR and sequencing primers. This contradictory result was unexpected. We further examined the sequence chromatogram of JPB34356 and found that its sequence contained many heterozygous sites (multiple signal peaks) in positions where the variable sites between unisexuals and clade B *A. barbouri *were located (Figure 2). At these heterozygous sites, *A. barbouri*'s sequence signals were generally weaker than unisexual-like sequence signals so the latter would be read by the program by default (Figure 2). We ruled out the possibility of DNA cross contamination and mitochondrial heteroplasmy in JPB34356 because, using primers GMM which targeted a much longer fragment for PCR amplification and sequencing, we consistently found a pure *A. barbouri *haplotype from JPB34356. In the sequence chromatogram, unisexual-like sequence signals were no longer detectable (Figure 2A). We used various primer combinations to amplify mitochondrial genes of JPB34342 using a new DNA extraction in the present study as well as an old DNA extraction used by Robertson et al. [19]. None of the amplicons showed any signs of DNA contamination or mitochondrial heteroplasmy. Additionally, through sequencing the mtgenome of *A. barbouri *JPB34342, unisexual JPB39932 and other specimens we showed that cyt-b gene did not have any duplicated component residing in the mitochondrial genome. A plausible explanation was that JPB34356 contained a nuclear copy (or copies) of a unisexual-like mitochondrial cyt-b gene fragment (*numt *or pseudogene). When using whole genomic DNA as template, both the actual mitochondrial cyt-b gene and its nuclear *numt *could have been co-amplified by MVZ15 and MVZ16. To further test this prediction we used the mitochondrial DNA fragment as a template and conducted sequencing PCR using MVZ15 and MVZ16 as primers. The results showed that no heterozygous sites existed in the sequence chromatogram (Figure 2A) and JPB34356 did not contain a unisexual-like cyt-b haplotype in its mitochondrial genome. Another primer combination using MVZ15 and MLM651 (targeted a ~2200 bp fragment too) was also used to amplify cyt-b using JPB34356 whole genomic DNA as template and no unisexual-like signals were detected in that sequence chromatogram.

### Mtgenome phylogeny

**Figure 3:**
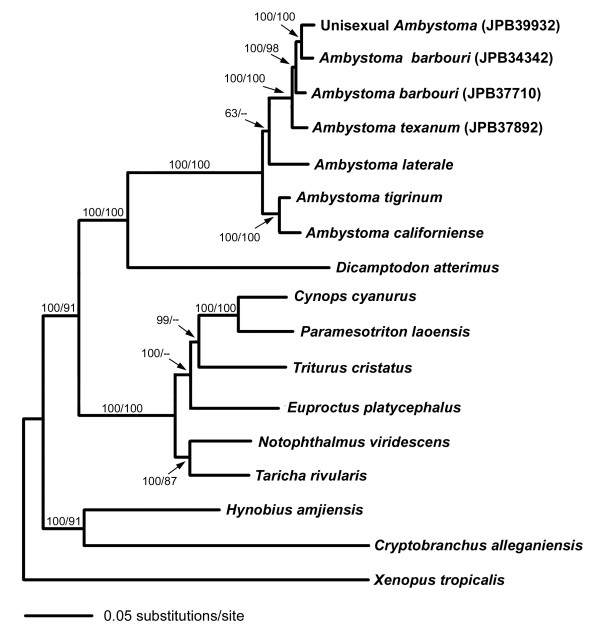
**A close phylogenetic relationship of unisexual *Ambystoma *(JPB39932) and Kentucky *A. barbouri *(JPB34342) inferred from mtgenome phylogeny**. Other species are incorporated to introduce calibration points for molecular dating analyses, and to serve as outgroups. Left numbers along branches represent the Bayesian posterior probabilities (BPP) and right numbers represent bootstrap proportions (BSP) derived from the parsimony analysis. BPP and BSP below 50 are not shown. Branch lengths are estimated by the Bayesian inference.

A total of 17 mtgenome sequences, including 13 downloaded from GenBank, were used for phylogenetic construction (Table 2). The combined DNA dataset contained 9647 bp with 3464 variable sites and 2361 were phylogenetically informative. Parsimony analysis generated a single tree 8803 steps in length (CI = 0.569, RI = 0.614). For Bayesian analysis, a GTR+I+G model was selected as the best-fit model. The tree topologies derived from parsimony and Bayesian analyses were similar, with slight differences in the relationships of some outgroup species, and we present the Bayesian tree in Figure 3. The monophyly of Kentucky *A. barbouri *JPB34342 and unisexual JPB39932 was strongly supported by both analyses (BSP = 100; BPP = 100).

**Table 2:** Species and unisexual biotype LJJJ used for mtgenome phylogeny and molecular dating analyses in this study with their GenBank accession numbers.

Species	Vouchers	GenBank Accession No.	References
*Ambystoma barbouri*	JPB34342	GU078469	This study
*Ambystoma barbouri*	JPB37710	GU078470	This study
*Ambystoma texanum*	JPB37892	GU078471	This study
Unisexual LJJJ	JPB39932	GU078472	This study
*Ambystoma californiense*	-	NC_006890	[43]
*Ambystoma la****t****erale*	-	NC_006330	[44]
*Ambystoma tigrinum*	-	NC_006887	[43]
*Cryptobranchus alleganiensis*	-	GQ368662	[45]
*Cynops cyanurus*	-	EU880309	[46]
*Dicamptodon atterimus*	-	GQ368657	[45]
*Euproctus platycephalus*	-	EU880317	[46]
*Hynobius amjiensis*	-	NC_008076	[47]
*Notophthalmus viridescens*	-	EU880323	[46]
*Paramesotriton laoensis*	-	EU880328	[46]
*Taricha rivularis*	-	EU880334	[46]
*Triturus cristatus*	-	EU880336	[46]
*Xenopus tropicalis*	-	NC_006839	JGI direct submission

The overall sequence divergence of mtgenome between the unisexual sample JPB39932 and *A. barbouri *JPB34342 was 4.42% (Table 3). We divided the mtgenome into 18 partitions and the pairwise difference of each partition ranged from 2.27% (concatenated tRNAs) to 7.03% (intergenic spacer). All 13 protein-coding genes were rather variable between genomes of JPB39932 and JPB34342. Except for ND4L (3.03%) and ATP8 (3.57%), the pairwise differences of all other genes were no less than 4.50%. JPB34342 was found to have an identical cyt-b haplotype to unisexuals by Robertson et al. [19]. Our study clearly demonstrated that neither the cyt-b gene nor any other genes, tRNAs or NCR throughout the mitochondrial genome were the same between *A. barbouri *JPB34342 and the unisexual.

**Table 3:** Sequence pairwise divergence of 18 genes/regions of mtgenome between the unisexual sample JPB39932 and *A. barbouri *JPB34342

Partitions	Sequence pairwise distances
Overall	4.42%
12s	2.60%
16s	2.70%
ND1	5.68%
ND2	5.56%
COI	5.43%
COII	5.10%
ATP8	3.57%
ATP6	4.53%
COIII	4.85%
ND3	5.98%
ND4L	3.03%
ND4	5.26%
ND5	4.62%
ND6	5.61%
CYT-B	5.17%
Intergenic spacer	7.03%
Control region	3.64%
tRNAs	2.27%

### Molecular dating

**Figure 4:**
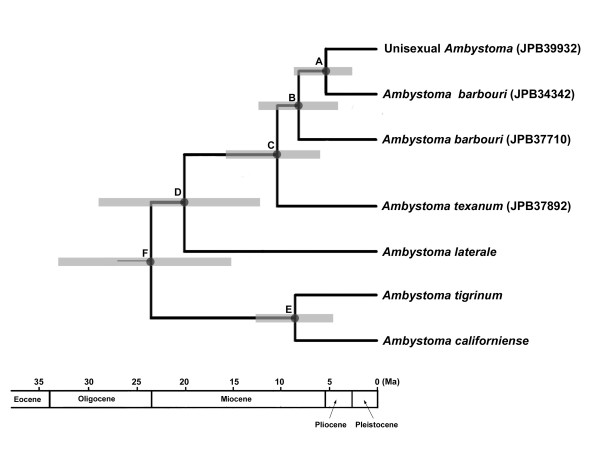
**A time-calibrated phylogeny inferring the origin of unisexual *Ambystoma*, fitted to a geological timescale**. Times for nodes [A-F] are estimated by BEAST. The horizontal gray bars through these nodes indicate 95% credibility intervals from the BEAST analyses. Non-ambystomatid specimens are not shown in the tree. Detailed time estimates can be found in Table 4. An early Pliocene origin for unisexual *Ambystoma *is proposed.

The relaxed lognormal clock analysis of the mtgenome sequences by BEAST produced the same topology as the Bayesian analysis. The divergence times estimated by BEAST and MultiDivTime are listed in Table 4. In general, the time estimates by BEAST and MultiDivTime were largely congruent. We present a time-calibrated tree from BEAST in Figure 4. The split between unisexual *Ambystoma *and Kentucky *A. barbouri *(Node A) took place about 5.3 Ma (CI 2.4, 8.7) by BEAST, and 5.1 Ma (CI 2.7, 9.0) by MultiDivTime. Both analyses agreed that the origin of the unisexual linage may date back to early Pliocene.

**Table 4:** Divergence time means and 95% confidence intervals calculated by BEAST and MultiDivTime*.

Nodes	BEAST	MultiDivTime
A:	5.3 (2.4, 8.7)	5.1 (2.7, 9.0)
B:	8.1 (4.4, 12.4)	7.5 (3.9, 13.1)
C:	10.4 (6.0, 15.4)	9.2 (4.9, 15.9)
D:	20.2 (12.4, 28.8)	21.0 (12.0, 32.8)
E:	8.6 (4.7, 12.8)	12.5 (6.5, 21.5)
F:	23.6 (15.1, 33.1)	23.5 (13.7, 36.0)

*Time unit is Ma (million years ago). The detailed description for each node can be found in Figure 4.

## Discussion

### No *Ambystoma barbouri *shares an identical mitochondrial cyt-b gene with unisexuals

The phylogenies generated using cyt-b, NCR as well as mtgenome all support the hypothesis that unisexual *Ambystoma *form a monophyletic group and share the most recent common ancestor with an *A. barbouri *lineage from Kentucky [5,9,19]. The age of unisexual *Ambystoma *was in high disagreement because four *A. barbouri *individuals (JPB34337, JPB34342, JPB34343, JPB34356) examined by two previous studies demonstrated distinctly different phylogenetic relationships to the unisexual lineage. Robertson et al. [19] found that these four *A. barbouri *were grouped with the unisexual cyt-b clade that suggested a very recent origin of unisexual lineage which was less than 25,000 years ago. On the other hand, Bogart et al. [5] found a 3.91% sequence pairwise distance in the control region between unisexuals and a few Kentucky *A. barbouri *individuals (including JPB34342, JPB34343, JPB34356) and claimed an ancient evolutionary history of unisexual *Ambystoma*, approximately 2.4-3.9 Ma. When using primers targeting a long mitochondrial fragment (~2200 bp), we found that no *A. barbouri*, including the four controversial *A. barbouri *individuals used by Robertson et al. [19], shared the same cyt-b gene sequence with unisexuals. The average cyt-b sequence pairwise distance between unisexuals (clade A) and their closest *A. barbouri *relatives (clade B) is as high as 5.16%. The examination of mtgenome of the representative specimens further demonstrated that neither the cyt-b gene, nor other genes/regions in the mitochondrial genome were identical or almost identical between unisexuals and *A. barbouri*. The overall substitution rates throughout the mitochondrial genome, especially in protein coding genes, were found to be rather consistent. Through a thorough sampling of *A. barbouri *across its distribution, Niedzwiecki (personal communication) did not find any haplotype that was more similar to the unisexuals than western clade Kentucky *A. barbouri *including specimens JPB34337, JPB34342, JPB34343 and JPB34356. Therefore, all our evidence clearly demonstrates that there is no known *A. barbouri *that shares an identical cyt-b gene with unisexuals.

### Molecular dating demonstrates an ancient origin of unisexual *Ambystoma*

In our BEAST analysis, the rate covariance among adjacent branches was 0.13 which was close to zero and their 95% confident intervals spanned zero (CI -0.20, 0.46). This result suggests that rate autocorrelation is insignificant [23] and BEAST (without rate-autocorrelation assumption), rather than MultiDivTime (with rate-autocorrelation assumption), would be more appropriate for the data and calibration choices in our study. Nevertheless, our results show that the time estimations between BEAST and MultiDivTime are largely congruent, especially within the ambystomatid salamanders (Table 4). Both analyses show that unisexual *Ambystoma *and Kentucky *A. barbouri *descended from their most recent common ancestor about 5 Ma so this dichotomy likely occurred in early Pliocene. More conservatively, if we take the lowest value at 95% CI inferred by both analyses, the unisexual lineage originated at least 2 Ma (early Pleistocene). Generally, our estimate was consistent with previous time estimates of the origin of unisexual *Ambystoma *[5,20,21] which validates them as the most ancient unisexual vertebrate lineage known to exist [2].

Niedzwiecki [22] suggested that the Kentucky and Ohio Rivers likely act as barriers between western and northern *A. barbouri *mtDNA clades (corresponding respectively, to clades B and C in our study, Figures 1B &1C). The Kentucky River might have been responsible for the divergence between *A. barbouri *from *A. texanum *as well as for the divergence of geographically distinct *A. barbouri *mtDNA lineages. Using a rough calibration approach (1.5% per million years for the *A. tigrinum *complex) [24], Niedzwiecki [22] estimated that various *A. barbouri *mitochondrial lineages were separated around 3.5-5 Ma and a similar time would have elapsed since the split between the main *A. barbouri *clades and *A. texanum. *Our study suggests that both divergence times are possibly more ancient than these estimates. In the BEAST analysis, for example, the separation between Kentucky *A. barbouri *(represented by clade B, Figures 1B &1C) and Ohio *A. barbouri *(represented by clade C, Figures 1B &1C) likely took place 8.1 Ma (CI 4.4, 12.4). The split of western and northern *A. barbouri *from *A*. *texanum *likely occurred 10.4 Ma (CI 6.0, 15.4). Our results indicate that both events may have taken place in late to middle Miocene or at least in early Pliocene, suggesting a longer evolutionary history for *A. barbouri *than expected.

### Is a unisexual-like cyt-b *numt *present in *A. barbouri*?

Nuclear copies of mitochondrial genes or *numts*, which evolve independently as paralogous copies of the original mitochondrial DNA segment, are relatively common and have been reported in various taxa [25-30]. Failure to discriminate real mitochondrial sequences and *numts *often confuses the genetic diversity of species and produces erroneous phylogenies [31]. Because the nuclear genome has an overall slower mutation rate than the mitochondrial genome, estimates on divergence times can be problematic [28,32]. *Numts *can originate from all parts of the mitochondrial genome including the cyt-b gene [33] and the majority of *numts *are fairly short sequences [34].

When using whole genomic DNA as template and primers MVZ15 and MVZ16 for PCR amplification and sequencing [19], *A. barbouri *individual JPB34356 possessed two different PCR products. One of the two was not detectable in the sequence chromatograms when using mitochondrial DNA as template with MVZ15/MVZ16 as sequencing primers, or using total genomic DNA as template with other various primer combinations. DNA cross contamination can yield sequence heterozygosity but contamination should have affected each gene/region across the entire mtgenome. We tested the old DNA extractions of *A. barbouri *individuals (JPB34337, JPB34342, JPB34343, JPB34356) used by Robertson et al. [19], and we did not find any signs of DNA contamination in any other genes/regions. Mitochondrial heteroplasmy may also be responsible for mixed mitochondrial amplicons [35,36]. The possibility that these *A. barbouri *individuals embrace *A. barbouri *and complete unisexual-like mtgenomes is minimal as proven by the experiments we conducted above. Mitochondrial heteroplasmy could also be caused by regional mutations or duplications of some mitochondrial copies [37,38]. If these *A. barbouri *individuals have two types of mtgenomes which are heteroplasmic only in the cyt-b gene, we should have detected the heterozygous signals in the cyt-b gene when using old/new total genomic DNA extractions or mitochondrial DNA as templates with any primer combinations for PCR and sequencing. The most reasonable explanation is that *A. barbouri *individual JPB34356 contains a unisexual-like cyt-b *numt*. This unexpected discovery in JPB34356 has resolved the mystery and provides a reasonable explanation for the data that were obtained by Robertson et al. [19]. We believe that a unisexual-like cyt-b *numt *was present in some *A. barbouri *individuals, including JPB34337, JPB34342, JPB34343, and JPB34356. It is very possible, in their study, that the cyt-b sequences recovered from these four *A. barbouri *were in fact unisexual-like *numts*. Therefore, the subsequent phylogenetic construction and divergence time estimates that were based on these putative *numts *were erroneous. Although we did not detect stop codons in these *numts*, it is known that *numts *do not always include stop codons or indels and sometimes are indistinguishable to their mitochondrial orthologs [35]. It is possible, however, that the stop codons were situated up/downstream beyond the region amplified by the MVZ15/MVZ16 primers that we used. Whether *numts *can be amplified may also depend on the quality and quantity of nuclear DNA template in the PCR reaction. In our study we failed to find or amplify the same sequences in three other controversial specimens JPB34337, JPB34342 and JPB34343, which likely resulted from the more highly degraded nuclear genomic DNA from these samples.

### Implications of having an ancestral cyt-b *numt *in *A. barbouri*

Dating the origin of unisexual/asexual lineages is usually based on the extent of genetic divergence from their nearest sexual relatives [7,39]. In general, time estimates are more robust if both mitochondrial and nuclear genomic markers are used as inferences. Uncoupling evolutionary trajectories between their mitochondrial and nuclear genomes, however, make molecular dating of unisexual *Ambystoma *depend solely on the mitochondrial genome. All unisexual *Ambystoma *share one mitochondrial origin but the evolutionary histories of their nuclear genomes are much more complicated and dynamic. It is clear that no recurrent hybridization between any of the sexual sperm donors produces new unisexual lineages [5,9] otherwise unisexual individuals would contain a mitochondrial genome derived from one or more of the extant sexual, sperm - donating species of *Ambystoma*. Bogart et al. [15] showed that *A. barbouri *can serve as a sperm donor for unisexuals in a single pond from southern Ohio. The cyt-b and NCR haplotypes recovered from those *A. barbouri *were found to be grouped in clade C (Figures 1B &1C), and are distantly related to the unisexuals. Without recurrent hybridization, unisexual individuals have evolved an extremely flexible reproductive mode by which they rely on sexual sperm donors to perpetuate. Unisexuals replace their nuclear genomes with those from sexual sperm donors, historically and contemporarily, throughout their entire distribution [40]. Consequently, it is impossible to use nuclear genome sequences for molecular dating of unisexual *Ambystoma*.

Little and Hebert [41] have criticized the traditional dating strategy used for unisexual and asexual organisms [39] because dating, not only requires a thorough search of extant taxa, but also related species that may have gone extinct since unisexuality/asexuality originated. Neiman et al. [42] reviewed asexual lineage longevity among invertebrate and vertebrate taxa and criticized the notion that there existed young and "ancient" asexual lineages. They found that the distribution of asexual lineages followed a regular and linear age distribution. The tabulated ages of asexual vertebrate lineages (fish, amphibians, and reptiles) were all less than 300,000 years old and could even be considered young compared with the estimated age of a sexual species. The unisexual *Ambystoma *lineage was included in their table of asexual lineages with an age <25,000 years based on the incorrect time estimate provided by Robertson et al. [19]. Our present study demonstrates that unisexual *Ambystoma *have persisted for about 5 million years and we attribute this longevity to the fact that they are unisexual, not asexual, and have a unique reproductive mode (kleptogenesis)[5].

Because *A. barbouri *may have an ancient evolutionary history [[22], this study], it is reasonable to assume that there may have existed a closer common ancestor to the unisexuals which became extinct at some unknown time [5]. Finding a unisexual-like cyt-b *numt *in some *A. barbouri *individuals suggests that its origination must have taken place at about the same time as the hybridization event that gave rise to unisexuals. Once a *numt *is established in its descendants' nuclear DNA, it would be much more conserved than the mitochondrial haplotype because the mitochondrial cyt-b mutates faster than its *numt*. The presence of an ancestral cyt-b *numt *in the western Kentucky clade of *A. barbouri *would also suggest that no other population of *A. barbouri*, including any potential extinct lineages, needs to be considered more closely related to the unisexuals.

## Conclusions

Using multiple sets of empirical evidence and rigorous statistical methodologies, we reject the conclusion of a recent origin of unisexual *Ambystoma *and support the hypothesis that unisexual *Ambystoma *is the most ancient lineage in unisexual vertebrates known to exist. The amplification of unisexual-like cyt-b sequence in a few Kentucky *A. barbouri *individuals is the possible cause of the erroneous phylogeny and time estimate determined by Robertson et al. [19]. The unisexual-like cyt-b sequence, presumably a *numt*, in a few Kentucky *A. barbouri *could represent a useful molecular "fossil" showing that the *A. barbouri *lineage from Kentucky represents the closest relatives to the unisexuals. Interestingly, our study shows that the unisexuals' mitochondrial genomes, which are descended from ancestral *A. barbouri*, seem to have changed little over time because their cyt-b genes are very similar to the ~800 bp ancestral cyt-b *numt *amplified sequence identified in some extant *A. barbouri *individuals. Low mitochondrial DNA variation among present unisexual populations have been described before [5,20] and it might be attributed to population bottlenecking of unisexuals prior to their rapid expansion after the Last Glacial Maximum. The length, number of copies and distribution of the cyt-b *numt *in *A. barbouri *is not yet known and there may be other *A. barbouri *individuals that have similar cyt-b *numts *and/or other ancient signatures shared with unisexuals. The confirmation of an ancient ancestry of unisexual *Ambystoma *validates them as an excellent model system for studying the evolution and maintenance of unisexuality in vertebrates.

## Methods

### Samples

*Ambystoma barbouri *from various populations from Kentucky, Ohio and Tennessee were examined. Because unisexuals were found to share a highly conserved mitochondrial genome in previous studies [5,9,19,20], we only sequenced a few unisexual individuals from several geographically distant populations to represent the unisexual lineage. The four specimens that were used for sequencing of their mtgenomes were: *A. barbouri *from Kentucky (JPB34342), unisexual LJJJ from Kentucky (JPB39932), *A. barbouri *from Ohio (JPB37710), and *A. texanum *from Ohio (JPB37892). To introduce calibration points into the molecular dating analyses, mtgenome sequences of 12 salamander species were retrieved from GenBank [43-47]: *A. californiense*, *A. laterale*, *A. tigrinum*, *Cryptobranchus alleganiensis*, *Cynops cyanurus*, *Dicamptodon atterimus*, *Euproctus platycephalus*, *Hynobius amjiensis*, *Notophthalmus viridescens*, *Paramesotriton laoensis*, *Taricha rivularis*, and *Triturus cristatus*. A frog, *Xenopus tropicalis*, was used as an outgroup. Its mtgenome was also downloaded from GenBank. Detailed sampling and sequence information is listed in Tables 1 and 2.

### Laboratory protocols

Total genomic DNA was extracted from frozen larvae, adult muscle, heart or liver tissues using a standard phenol-chloroform extraction method. We also tested some DNA extractions used by previous studies [5,19]. A combination of 22 primers [45] was used to amplify contiguous and overlapping fragments that covered the entire mitochondrial genome. PCR was conducted in a 25 μl mix including 30-50 ng of template DNA, 1 U Taq DNA polymerase (TaKaRa), 1× PCR buffer, 1.5 mM MgCl_2_, 0.2 mM of each dNTP, and 10 pmol of each primer. PCR cycling parameters were 95°C for 5 min as initial denaturation, followed by 30 cycles of 95°C for 30 sec, 45-50°C [45] for 45 sec, 72°C for 30 sec to 2 min depending on the expected size of fragments (approx. 1 min/kb), and a final step at 72°C for 5 min. PCR products were verified on 1% agarose gels and purified using QIAquick PCR purification kits (Qiagen). Sequencing was performed with the corresponding PCR primers using BigDye 3.1 terminator sequencing chemistry with an ABI 3730 (Applied Biosystems). Sequences were assayed using Sequencher (version 4.5; Gene Codes). For some large PCR fragments that were more than 1600 bp, specific internal primers were designed to obtain their complete sequences.

Primers Glu14100L [45] and MLM651 [24] were used to amplify a ~2200 bp mitochondrial fragment that included cyt-b, intergenic spacer and control region. An internal primer M2R (reverse primer, 5'-GTTGGTGGTTTCTCGCCCTAAG-3') was designed for sequencing across the complete fragment. As a comparison, we also used the same primers MVZ15 and MVZ16 [48] that were used to amplify cyt-b genes by Robertson et al. [19] to examine some of the *A*. *barbouri *individuals, especially those that had an identical or almost identical cyt-b haplotype to unisexuals. These two primers targeted an ~800 bp partial cyt-b gene fragment [19]. Likewise, we used the same primers MLMTHR and MLM651 [24] that were used by Bogart et al. [5] to amplify NCRs from some specimens and the expected size of PCR products was approximately 1100 bp [5]. The PCR annealing temperature was set to 50°C for MVZ15 and MVZ16, and was set to 46°C for MLMTHR and ML651. The protocols of standard PCR reaction, purification, sequencing and sequence alignment were the same (above).

### Phylogenetic methods

Three phylogenies were constructed using mtgenome, cyt-b, and NCR, respectively. When using mtgenomes for phylogenetic construction, we excluded all the tRNAs and non-coding regions and only included two rRNAs and 13 protein-coding genes. This was necessary because of the absence of several tRNAs and the lack of the intergenic spacer in mtgenomes of some outgroup species as well as the difficulty in sequence alignment of control regions. With the 13 protein-coding genes, the third codon positions were eliminated because of high substitution rates. Multiple substitutions likely produce noise in phylogenetic and dating analyses [45]. Ambiguous alignments in the two rRNA regions were also excluded. Additionally, all the gaps in the alignments were eliminated manually. Finally, a DNA dataset containing all 17 DNA alignments (each with two rRNAs and 13 protein-coding genes without third codon positions) was generated.

A maximum parsimony analysis was conducted using PAUP* (version 4.01b10) [49]. Each sequence was treated as a taxon and each nucleotide was treated as a character. All characters were weighted equally and unordered. A heuristic search method via tree-bisection-reconnection (TBR) branch swapping was used. Bootstrap proportions (BSP) [50] with 1,000 replicates were used to evaluate the nodal support. A Bayesian analysis was conducted using MrBayes (version 3.1) [51]. Model selection was based on the Akaike information criterion (AIC) as implemented in MrModeltest (version 2.2) [52]. The best-fit model was used in subsequent Bayesian phylogenetic analysis. Four Markov chains were used and the dataset was run for 10,000,000 generations to allow adequate time for convergence. Trees were sampled every 500 generations, and the last 5,000 trees were used to estimate the consensus tree and the Bayesian posterior probabilities (BPP). The overall sequence pairwise divergence between mtgenome of Kentucky *A. barbouri *and the unisexual was calculated using MEGA (version 4.0) [53]. The mtgenomes were then divided into 18 partitions according to genes and regions (one concatenated tRNAs, two rRNAs, two non-coding regions and 13 protein-coding genes) and sequence pairwise difference of each partition between Kentucky *A. barbouri *and unisexual respectively, was calculated. Pairwise differences of cyt-b and NCR between the major lineages of interest were also calculated by MEGA.

### Molecular dating

To infer the TMRCA for the unisexual lineage and its closest relative, Kentucky *A. barbouri*, we incorporated 13 previously published mtgenome sequences to allow the calibration points to be introduced to our analysis. A combined DNA dataset including two rRNAs and 13 protein-coding genes without third codon positions were used for dating analyses. Gaps in the DNA alignment were manually excluded. The ingroup root of the tree (Salamandroidea and Cryptobranchoidea) was constrained to be 151 to 170 Ma. This constraint was based on the oldest salamander fossil known to exist, the salamandroid-like *Iridotriton hechti *(151 Ma) [54] and a proposed maximal bound for the origin of Caudata (170 Ma) [55]. The *Taricha*-*Notophthalmus *split was constrained to be at least 23 Ma (*Taricha oligocenica *from the upper Oligocene) [56]. The *Ambystoma*-*Dicamptodon *split was set to at least 55.8 Ma (*Dicamptodon antiquus *from the late Paleocene) [57]; The *Euproctus *-*Triturus *split was set to 20-30 Ma (disjunction of the Corsica-Sardinia microplate from the Iberian Peninsula) [46]; The *Cynops*-*Paramesotriton *split was constrained to be greater than 15 Ma (*Procynops miocenicus *from the upper Miocene) [56]. The split of *A*. *tigrinum*- *A*. *californiense *was set to be around 5 Ma (the beginning of Sierran uplift) [24]. Because most of the fossil record we used in this study only provided a minimum age for the origin, when setting the priors we used the fossil ages as the lower bounds.

Molecular dating was conducted by a Bayesian MCMC approach in the program BEAST (version 1.5.1) [58] and MultiDivTime [59]. Using BEAST, the topology and divergence times can be estimated simultaneously from the data. BEAST input files were generated with BEAUTi (version 1.5.1). A GTR+I+G was used to describe the substitution model, a Yule process was used to describe speciation, and an uncorrelated lognormal (UCLN) model was used to describe the relaxed clock [23]. We used a lognormal distribution for fossil calibrations, and a normal distribution for the biogeographic calibrations. BEAST was run for 80,000,000 generations with samples taken every 1,000 generations. Five independent MCMC runs were conducted and the log and time tree files were combined using LogCombiner (version 1.5.1). The results were examined by Tracer (version 1.4.1) to confirm stationary distribution and adequate effective sample sizes (ESS) that had been obtained for all parameters. TreeAnnotator (version 1.5.1) was then used to summarize a best-supported tree and annotate the tree with the mean age and posterior probabilities of the nodes under investigation. FigTree (version 1.2.3) was used to display the estimated tree with node ages and the 95% confidence intervals. Programs BEAST, BEAUTi, LogCombiner, Tracer, TreeAnnotator and FigTree were downloaded from http://beast.bio.ed.ac.uk. In the MultiDivTime analysis, parameters of the substitution model were first estimated by program Baseml in the PAML package (version 4.3) [60]. The output from Baseml was then used in the Multidistribute package to estimate the maximum likelihood of the branch lengths and a variance-covariance matrix, and to perform a MCMC Bayesian analysis for estimating the posterior distributions of substitution rates and divergence dates. The tree presented in Figure 3 was used as the reference topology for molecular dating analysis. The priors for the ingroup root age mean (rttm) and standard deviation (rttmsd) (Salamandroidea - Cryptobranchoidea split, 151-170 Ma) were set to 1.60 and 0.1, respectively. The mean and standard deviation of the prior distribution for the rate of molecular evolution at the ingroup root node (rtrate and rtratesd) were both set to 0.12. The prior mean and standard deviation for the Gamma distribution of the parameter controlling rate variation over time (i.e. brownmean and brownsd) were both set to 0.5. The Markov chain was run for 1,000,000 generations and sampled every 100 generations with an initial burn-in of 200,000 generations. Three independent runs were performed to ensure the convergence. The PAML package was downloaded from http://abacus.gene.ucl.ac.uk/software/paml.html, and the Multidistribute package from http://statgen.ncsu.edu/thorne/multidivtime.html.

## Authors' contributions

KB detailed the experimental design and performed most of the lab work, data analyses and manuscript preparation. JPB conceived and directed the study. Both authors contributed equally to this work in discussing research strategy and development. Both authors read and approved the final manuscript.
